# Self-regulated oscillation of transport and topology of magnetic islands in toroidal plasmas

**DOI:** 10.1038/srep16165

**Published:** 2015-11-04

**Authors:** K. Ida, T. Kobayashi, T. E. Evans, S. Inagaki, M. E. Austin, M. W. Shafer, S. Ohdachi, Y. Suzuki, S.-I. Itoh, K. Itoh

**Affiliations:** 1 National Institute for Fusion Science, Toki, Gifu 509-5292, Japan; 2 General Atomics, San Diego, California 92186-5608, USA; 3 Research Institute for Applied Mechanics, Kyushu Univ., Kasuga, 816-8580, Japan; 4 University of Texas, Austin, Texas 78712, USA; 5 Oak Ridge National Laboratory, Oak Ridge, Tennessee 37831, USA

## Abstract

The coupling between the transport and magnetic topology is an important issue because the structure of magnetic islands, embedded in a toroidal equilibrium field, depends on the nature of the transport at the edge of the islands. Measurements of modulated heat pulse propagation in the DIII-D tokamak have revealed the existence of self-regulated oscillations in the radial energy transport into magnetic islands that are indicative of bifurcations in the island structure and transport near the *q* = 2 surface. Large amplitude heat pulses are seen in one state followed by small amplitude pulses later in the discharge resulting in a repeating cycle of island states. These two states are interpreted as a bifurcation of magnetic island with high and low heat pulse accessibility. This report describes the discovery of a bifurcation in the coupled dynamics between the transport and topology of magnetic islands in tokamak plasmas.

In magnetized plasmas, isolated magnetic structures, which are characterized by locally closed magnetic surfaces and bounded by a magnetic separatrix, are self-organized in wider circumstances. Examples include magnetic islands in magnetically confined plasmas^1^, plasmoids in space plasmas^2^, stellar mass-ejection events^3^, and others. The structure occupies a small part in volume compared to the plasma system, but has an essential impact on the evolution of the whole plasma system. For instance, magnetic islands in magnetically confined plasmas are considered to play key roles in the onset of disruption^1^, and in the suppression^4^ and mitigation^5^ of edge localized modes (ELMs) by using resonant magnetic perturbation (RMP) fields.

Plasmoids strongly influence the interaction between space plasmas and the solar wind^2^. Magnetic islands, which are important in the nonlinear evolution of magnetized toroidal plasmas, evolve following the plasma dynamics. Stochastization of the magnetic-field, i.e. magnetic braiding or the appearance of the secondary magnetic island^6^,^7^,^8^ can occur when the island size reaches a critical value. In this case, an enhancement of the cross-field transport in the magnetic island has been observed in^9^,^10^. On the other hand, owing to the closed magnetic field line within the magnetic island, the pressure gradient is often eliminated within the islands so that turbulent transport can be suppressed in the magnetic island. These competing effects have been studied experimentally. A significant reduction of the particle transport was observed inside magnetic islands in PBX^11^ and JET^12^,^13^, which has been called a snake. The reduction of heat transport inside a magnetic island has been observed in both the Large Helical Device (LHD)^14^ and the JT-60U tokamak^15^. In some cases, the electron heat diffusivity inside the magnetic island was evaluated to be comparable with the transport in the ambient plasma^16^. At the same time, plasma transport inside an island can influence the evolution of magnetic island^17^. Thus, the study of transport properties inside the magnetic islands is an urgent issue in the study of high temperature plasmas.

Here we find self-regulated oscillations of transport and topology of magnetic island; one is a magnetic island with a high heat pulse accessibility due to turbulence penetration and the other is a magnetic island with low accessibility due to turbulence screening.

## Results

### Experimental setup

A key technique to be featured in the experiment in this paper is modulated electron cyclotron heating (MECH), which has been applied in LHD^9^,^18^ to study the change of magnetic topology, i.e., nested flux surfaces, small isolated magnetic islands, mixed islands and stochastic layers and regions of strong stochasticity, due to intrinsic resonant magnetic fields and applied RMP fields. The MECH experiments were recently carried out in the DIII-D tokamak^19^ where the size and phase of the magnetic island can be controlled by an external perturbation coil referred to as the C-coil^20^.

In this experiment, an *n* = 1 perturbation magnetic field is applied in DIII-D discharge 154526 to produce a large *m*/*n* = 2/1 non-rotating magnetic island at a normalized minor radius *ρ* = 0.64–0.8 with the C-coil amplitude of 3.35 kA, where *m* and *n* are the poloidal and toroidal mode numbers of the island respectively. A phase flip in the C-coil between 5° and 185° phases is performed. As a result of this phase flip, the X-point and O-point of the magnetic island appears, respectively, at the toroidal angle of electron cyclotron emission (ECE) measurement. The electron cyclotron heating (ECH) power is deposited near the *q* = 1 surface at *ρ* = 0.42 with a modulation frequency of 50 Hz. This operation contributes to the reduction of the sawtooth amplitude and frequency causing the amplitude of the sawtooth to be smaller than the MECH amplitude with a frequency that is less than 50 Hz or almost no sawtooth for several MECH periods.

### Observation of Self-regulated oscillation of magnetic topology

**Figure 1:**
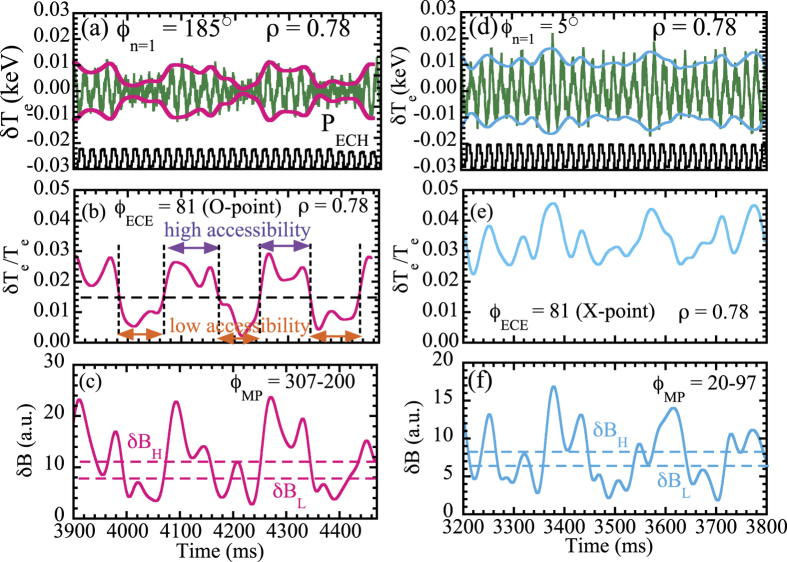
Time evolution of (**a**) electron temperature measured with ECE with a high pass filter (*f* > 30 Hz), *δT*_*e*_, and a series of ECH pulses, *P*_*ECH*_, (**b**) relative modulation amplitude of electron temperature, *δT*_*e*_/*T*_*e*_, using a low pass filter (*f* < 40 Hz) at *ρ* = 0.78 and (**c**) the modulation amplitude of the difference between the magnetic field measured with two magnetic probes, *δB*, located at the toroidal angle of 200° and 307° with the C-coil toroidal angle set at 185°, where the ECE diagnostics located at a toroidal angle with 81° (at O-point of magnetic island) and (**d**) *δT*_*e*_ and *P*_*ECH*_ (**e**) *δT*_*e*_/*T*_*e*_, and (**f**) *δB* at toroidal angle of 20° and 97° for the C-coil toroidal angle set for 5°, where the ECE diagnostics located at X-point of magnetic island. The dashed lines in (**e**,**f**) indicate the threshold for the conditional averaging of the low heat pulse accessibility state and high accessibility state, respectively. Shot number 154526.

Figure 1 shows the time evolution of (a,d) electron temperature measured with ECE with a high pass filtered (*f* > 30 Hz) channel located at *ρ* = 0.78, *δT*_*e*_, and a series of ECH pulses, *P*_*ECH*_, (b,e) relative modulation amplitude of electron temperature, *δT*_*e*_/*T*_*e*_, with a low pass filter (*f* < 40 Hz) at *ρ* = 0.78. In Fig. 1(c,f) the modulation amplitude of the difference between two magnetic field probes, separated by 107° and 77° Fig. 1(c) and Fig. 1(f) 77 degrees toroidally, is shown, *δB*. Figure 1(a–f) are for the C-coil toroidal phase of 185° and 5°, respectively. The ECE diagnostics located at the toroidal angle of 81° give the radial profiles of electron temperature along the O-point and X-point of the magnetic island for the C-coil toroidal phase of 185° and 5°, respectively. The modulation amplitude of *B*(*ϕ*_*MP*_ = 200°) − *B*(*ϕ*_*MP*_ = 307°) in Fig. 1(c) is larger than that of *B*(*ϕ*_*MP*_ = 20°) − *B*(*ϕ*_*MP*_ = 97°) in Fig. 1(f), because of the difference in toroidal angle distance of 107° and 77°.

The electron temperature measured with ECE shows a clear 50 Hz modulation with the amplitude of 5–10 eV associated with the ECH pulse, as seen in Fig. 1(a). An important finding is that the modulation amplitude oscillates with a frequency of 5 Hz, although the ECH modulation and the phase of the C-coil is constant. The modulation envelope is evaluated from the amplitude of fundamental component (50 Hz) of the ECE signal using a running FFT analysis. There are two states of modulation amplitude: one is with large modulation amplitude and the other is small modulation amplitude. The former corresponds to the magnetic island with high heat pulse accessibility and the later corresponds to the magnetic island with low heat pulse accessibility. Figure 1(b) shows the time evolution of the relative modulation amplitude, 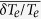. The relative modulation amplitude is 2–3% in the magnetic island with high accessibility, while it is less than 1% in the magnetic island with low accessibility. Two states of the magnetic island (high and low heat pulse accessibility) are clearly observed both in the temperature at the magnetic island and magnetic field *B*(200°) − *B*(307°) measured with two probes located at the low field side for the C-coil toroidal phase of 185°. The ECE diagnostics is located at O-point of magnetic island. On the other hand, when the C-coil toroidal phase is 5°, where the ECE diagnostics is located at X-point of magnetic island, the two states of magnetic island (high and low heat pulse accessibility) are not clearly observed in the ECE signal. However, the modulation amplitude of difference of magnetic field, *B*(20°) − *B*(97°), measured with the magnetic probe pair located at the same toroidal angle respect to the C-coil toroidal phase clearly shows the existence of two states of magnetic island. Therefore the magnetic fields measured with two magnetic probes are used for the conditional averaging of the two sates for each toroidal phase of C-coil of 185° and 5° (3900–4470 ms and 3200–3800 ms). The thresholds for the conditional averaging of the high and low accessibility states are set by *δB*_*H*_ and *δB*_*L*_, i.e., *δB* > *δB*_*H*_ for the high accessibility state and *δB* < *δB*_*L*_ for the low accessibility state, respectively, as indicated with the dashed lines in Fig. 1(c) and Fig. 1(f).

**Figure 2:**
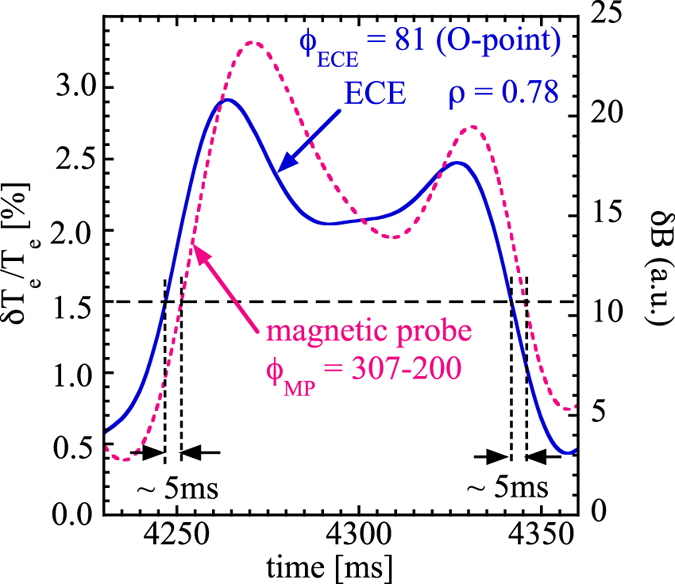
Expanded view of the relative modulation amplitude of electron temperature, *δT*_*e*_/*T*_*e*_, using a low pass filter (*f* < 40 Hz) at *ρ* = 0.78 and the modulation amplitude of the difference between the magnetic field measured with two magnetic probes, *δB*, located at the toroidal angle of 200° and 307° with the C-coil toroidal angle set at 185°, where the ECE diagnostics located at a toroidal angle with 81° (at O-point of magnetic island).

The modulation amplitude of electron temperature at the O-point of the magnetic island is well correlated to the modulation amplitude of magnetic field *B*(200°) − *B*(307°). The modulation of electron temperature precedes the modulation of magnetic field by ~5 ms as seen in Fig. 2. This fact suggest that the transition of ECE signal is not due to the change in magnetic fluctuations. The change in the modulation amplitude of the magnetic probe is considered to be the result rather than the cause of change in turbulence transport.

**Figure 3:**
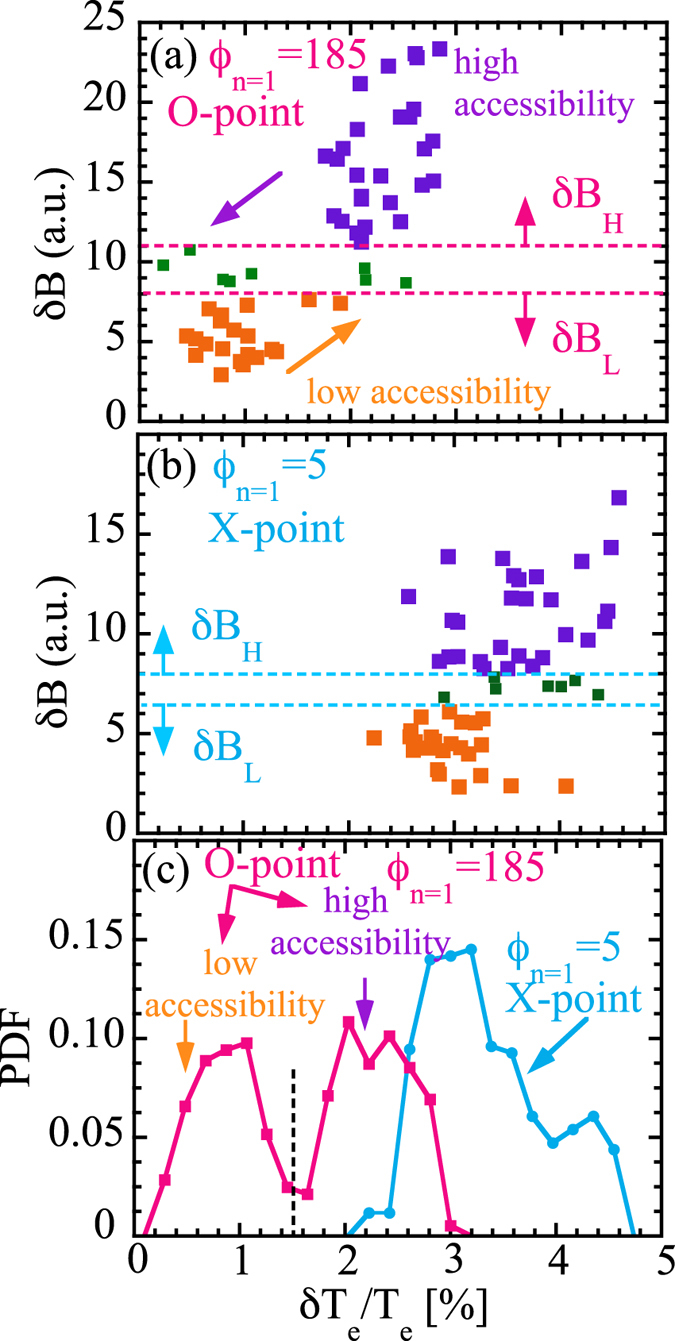
The relation between the modulation amplitude of the difference between the magnetic field oscillations measured with two magnetic probes, *δB* plotted in Fig. 1 and relative modulation amplitude of electron temperature at *ρ* = 0.78 with the C-coil toroidal phase angle set for (a) 185° (ECE located at O-point) and (b) 5° (ECE located at X-point) and (c) Probability distributed function of the heat pulse modulation amplitude as a function of *δT*_*e*_/*T*_*e*_ at *ρ* = 0.78 with the C-coil toroidal phase angle set for 185° (ECE located at O-point) and 5° (ECE located at X-point).

Figure 3(a,b) shows the relation between the modulation amplitude of the difference between the magnetic field oscillations measured with two magnetic probes, *δB* plotted in Fig. 1(c,f) and relative modulation amplitude of electron temperature at *ρ* = 0.78 with the C-coil toroidal phase angle set for 185° (ECE located at O-point) and 5° (ECE located at X-point) At the O-point, both the ECE and magnetic probe data can be divided to two groups either by the modulation amplitude of temperature (*δT*_*e*_/*T*_*e*_ > 1.5 % and *δT*_*e*_/*T*_*e*_ < 1.5%) or modulation amplitude of magnetic field (*δB* > *δB*_*H*_ and *δB* < *δB*_*L*_). At the X-point, the magnetic probe data spreads into the wide range similar to that at the O-point, however, the modulation amplitude of temperature is always high. In order to investigate the characteristics of heat pulse propagation for these two states, the relative modulation amplitude is evaluated by conditional averaging using ECH timing and *τ* is defined as time delay from the ECH off for the period of, *δB* > *δB*_*H*_ (a high accessibility state) and *δB* < *δB*_*L*_ (a low accessibility state). There are two quantitative measure to identify the two states: one is *δT*_*e*_/*T*_*e*_ and the other is *δB*. The *δT*_*e*_/*T*_*e*_ measurement is either at O-point or X-point and it is difficult to identify the state in the case of X-point, because the difference in two state is much smaller. Therefore we adopt *δB* as a quantitative measure to identify the time window of two states.

Figure 3(c) shows the probability distribution function of the modulation amplitude as a function of *δT*_*e*_/*T*_*e*_ at *ρ* = 0.78. Two clear peaks of PDF appear at the O-point of the magnetic island in the C-coil toroidal phase of 185°, which indicates that these two states are well separated and there is a bifurcation of the magnetic island dynamics separating the two states. In contrast, at the X-point of the magnet island in the C-coil toroidal phase of 5°, two clear peaks are not observed and the minimum in the PDF is above 2% which corresponds to the high heat pulse accessibility results for an island with broken outer flux surfaces. The signature for a low heat pulse accessibility is for the PDF to extend below ~1.5% in *δT*_*e*_/*T*_*e*_ as is seen in the O-point case. The most significant O-point low accessibility heat pulse PDF peak shown in Fig. 3(c) occurs in the ECE channel located at *ρ* = 0.78, just inside the boundary of the magnetic island, which indicates that this bifurcation phenomena occurs at the boundary of the magnetic island. There is no detectable difference in the radial profile of the mean electron temperature between these two states. This is because the spatial resolution of the ECE measurements, *δρ*_*ECE*_, is 0.04 and not enough to detect the difference in sharpness or discontinuity of radial profile of electron temperature.

**Figure 4:**
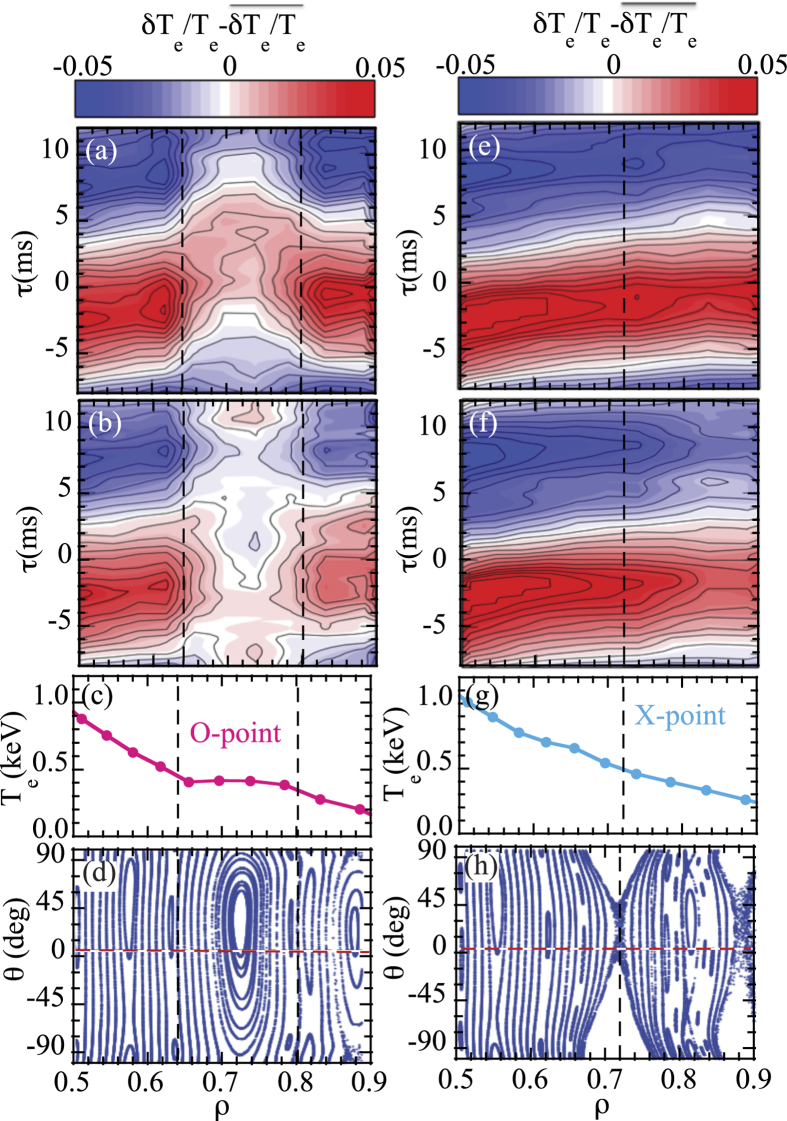
Contour of relative modulation amplitude of electron temperature in space and time for (a) high accessibility state and for (b) low accessibility state and (c) radial profiles of mean electron temperature and (d) the Poincaré map of the static vacuum magnetic field with RMP at O-point and the contour for (e) high accessibility state, and for (f) low accessibility state and (g) electron temperature profile and (h) the Poincaré map at X-point.

Figure 4 shows a contour of relative modulation amplitude of electron temperature in space and time for (a,e) the high accessibility phases and for (b,f) the low accessibility phase and (c,g) radial profiles of mean electron temperature, and (d,h) the Poincaré map at O-point and X-point calculated by vacuum magnetic field. In Fig. 4(d,h) the red dashed line at *θ* = 0° shows the poloidal position of the ECE measurements. Temperature flattening is clearly observed at the O-point in the region of *ρ* = 0.64–0.8, while no flattening of electron temperature is observed at the X-point. The width of the magnetic island evaluated by temperature profile is much larger than that expected by calculations with vacuum field of RMP using *n* = 1 C-coil current field. The width of the magnetic island can differ from that predicted by calculations due to the plasma response. The resistive MHD simulations predict either screening of the vacuum island or amplification depending on the magnitude of the perpendicular electron flow at the location of the island. When the flow is close to zero the simulations with M3D-C1 code predict an amplification^21^, which is consistent with the observations in this experiment where the magnetic island is not rotating due to the external C-coil perturbation field applied.

As seen in Fig. 4, the characteristics of heat pulse propagation shows clear differences between these two states at the O-point of the magnetic island. During the low accessibility phases, the modulation amplitude inside magnetic island is strongly reduced, while the reduction of modulation amplitude is weaker during the high accessibility phases. This is in contrast to the heat pulse propagation at the X-point, where there is no reduction of relative modulation amplitude near the X-point of the magnetic island. In Fig. 4(a,b,e,f), the relative modulation amplitude of the heat pulse starts to decrease after passing the X-point location in the low accessibility state, while the relative modulation amplitude is more or less constant in space in the high accessibility state. It is interesting that this bifurcation phenomena affects the relative modulation amplitude of the heat pulse propagation downstream of the *m*/*n* = 2/1 magnetic island (*ρ* > 0.8 at O-point and *ρ* > 0.72 at X-point). In the downstream region another magnetic island may appear, which will be discussed in future.

### Amplitude and delay time of the heat pulse

**Figure 5:**
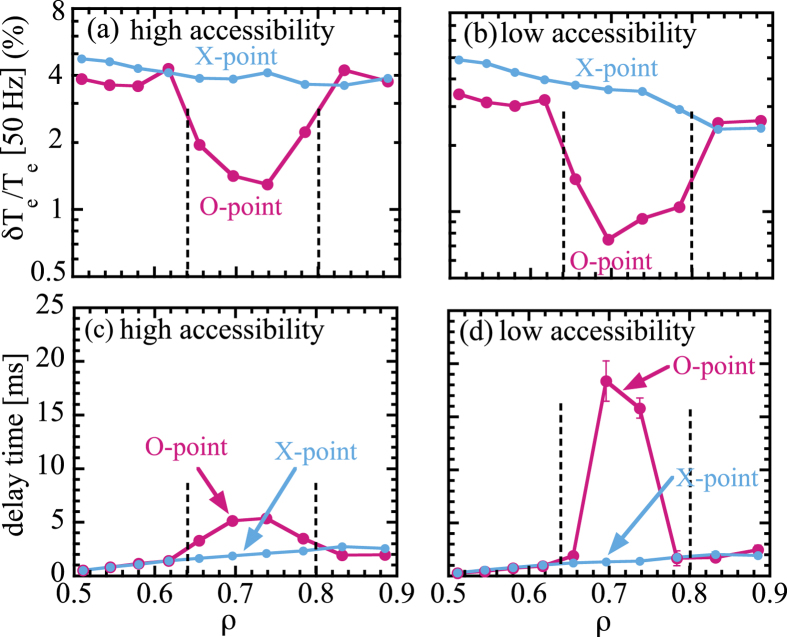
Radial profiles of modulation amplitude with fundamental frequency (50 Hz) for the (a) a high accessibility state (high accessibility magnetic island) and (b) a low accessibility state (low accessibility magnetic island) and the radial profile of delay time of heat pulse with fundamental frequency (50 Hz) for the (c) a high accessibility state (high accessibility magnetic island) and (d) a low accessibility state (low accessibility magnetic island).

After the conditional averaging discussed above, the fundamental frequency component of the heat pulse is extracted in order to eliminate the noise associated with the sawtooth crash, which usually appears as higher order components of modulation frequency. Figure 5 shows the radial profiles of modulation amplitude with fundamental frequency (50 Hz) for the (a) a high accessibility magnetic island and (b) a low accessibility magnetic island and the radial profile of delay time of heat pulse with fundamental frequency (50 Hz) for (c) a high accessibility magnetic island and (d) a low accessibility magnetic island. The modulation amplitude of the heat pulse decreases near the O-point of magnetic island, while there is no significant decrease of modulation amplitude at the X-point, which indicates a significant reduction of modulation amplitude inside the magnetic island. On the other hand, the delay time increases (and propagation speed slow down) near the O-point of magnetic, while there is no change in propagation speed in space at the X-point. A slow down of propagation speeds is observed both in the low and high accessibility magnetic island. The heat pulse propagation speed is extremely slow inside the magnetic island with low accessibility. This result suggests that there is significant reduction of transport inside the magnetic island and the magnitude of reduction of transport has a bifurcation; one is strong reduction of transport with a low accessibility and the other is moderate reduction of transport with a high accessibility.

The small modulation amplitude of the heat pulse is also consistent with the reduction of transport perpendicular to magnetic flux surface, because the modulation amplitude is determined by the balance between the heat pulse propagation speed parallel and perpendicular to the magnetic field line. The heat pulse propagates faster through X-point because of the reduction of perpendicular transport inside magnetic island. The transport inside the magnetic island is reduced by an order of magnitude compared to the X-point, which is consistent with the observation of lower thermal diffusively inside magnetic island than that outside by an order of magnitude in LHD^14^ and JT-60U^15^. In the low accessibility phase, the perturbation field with m/n = 2/1 component becomes dominant and produces magnetic island with low accessibility.

### Time scale of transition

**Figure 6:**
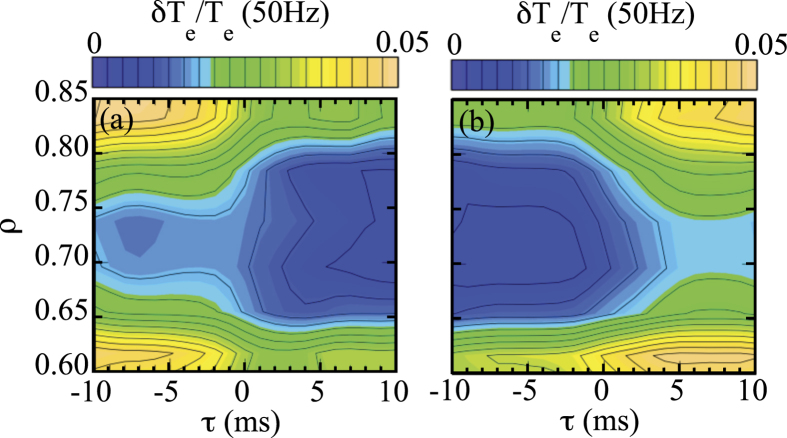
Contour of relative modulation amplitude of electron temperature in space and time during the (a) forward transition (from high accessibility to low accessibility magnetic island) and (b) backward transition (from low accessibility to high accessibility magnetic island) at O-point.

Figure 6(a) shows the contour of relative modulation amplitude of electron temperature in space and time during the transition from high accessibility (*τ* < 0) to low accessibility (*τ* > 0) magnetic island and Fig. 6(b) shows the back transition from low accessibility to the high accessibility magnetic island at O-point. Here, the timing of the conditional averaging is the zero crossing of the 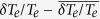 in Fig. 1(b) with negative slope for forward transition and with positive slope for backward transition. The region of the low relative modulation amplitude indicates the region with nested magnetic flux surfaces. Therefore, the magnetic island phase with a high heat pulse accessibility is interpreted as highly accessibility with a large edge accessible layer and a small region of nested flux surfaces inside the island.

This change in accessibility is expected to be related due to the change in transport with different magnitudes of turbulence just inside the magnetic island. Magnetic islands with low accessibility are interpreted as magnetic islands having a sharp magnetic island boundary due to the reduced transport just inside the magnetic island. In contrast, magnetic islands with high accessibility are interpreted as islands having a board boundary due to the enhancement of the transport just inside the magnetic island. The time scale of the forward transition is shorter (~4 ms) than that of backward transition (~7 ms).

## Working hypothesis

### Coupling of magnetic island and transport

The essence of experimental finding is the cross coupling between transport process and magnetic topology. According to the working hypothesis, when the penetration of heat pulse into magnetic island changes, the amplitude of resonant magnetic perturbation can change. The coupling between the island width and transport can be modeled by the Rutherford equation (e.g.,^17^.)


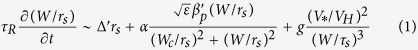



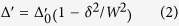


in the absence of relative rotation velocity between plasma and magnetic island, where *W* is the island width, 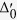 (negative in this case) is the tearing-stability parameter, *δ* is the island width due to the vacuum external magnetic field, indicating the induction of island by external coil^22^,^23^. The 2nd term on the RHS denotes the enhancement of island width by the lack of bootstrap current near the O-point of the island. *W*_*c*_ denotes the critical width of island, above which the neoclassical tearing instability mechanism works. (alpha is of the order unity). The 3rd term on the RHS, the ion-polarization current effect, is effective in the small *W* limit, but is ineffective in determining the saturated island width in the present experiment. This model equation has been discussed in conjunction the finite plasma pressure effect on the tearing mode, causing the coupling between transport and island dynamics. The critical width *W*_*c*_ is determined by the competition between parallel thermal conductivity and perpendicular thermal conductivity. It was given in^24^ as,


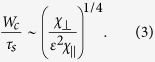


If the perpendicular thermal diffusivity, *χ*_⊥_, is large, the temperature gradient penetrates into the island, so that the Bootstrap current remains in the island, making the island thinner. As seen in Fig. 3(c,d), the width of the observed magnetic island is lager than that calculated by the vacuum magnetic field.

### Enhanced transport in the magnetic island

There are two typical states for the transport in the magnetic island. One is where the perpendicular thermal conductivity is small (as has been measured in^14^). The other is the case where the perpendicular transport is finite and *W*_*c*_ is not negligible in comparison with the island width *W*. In the latter case, an increment of perpendicular conductivity in the magnetic island can occur via two processes. One is the spreading of turbulence^25^,^26^ into magnetic island. The other is the onset of stochasticity.

These two processes of increasing the transport coefficient in island can result in two feedback paths of plasma response on the island width. The first is a turbulence spreading mechanism. This is prominent, when the island width is comparable to twice of penetration depth of turbulence into linearly-stable regime, *d*_*sp*_, where the perpendicular thermal conductivity in the island is enhanced and *W*_*c*_ increases so as to reduce the island width following Eq. (1). The smaller the island, more effective the penetration of turbulence. Thus, two states are possible: one is an enhanced island without the penetration of turbulent transport, and the other is the thin island where turbulence has tunneled into the island. In this link of dynamics, if the transition from one state to the other occurs, the a delay is predicted to occur in the magnetic response. The change in transport occurs, together with *W*_*c*_, then *W* evolves according to Eq. (4). The delay time is on the order of the magnetic diffusion time into the magnetic island. The second possible factor is the onset of stochasticity when the island width reaches the critical size. If the effective thermal conductivity is enhanced by stochastization (as has been discussed on LHD), *W*_*c*_ is enhanced so that the island width reduces. The reduction of island width stops stochastization.

### Self-organized chain

There are at least two self-organized links in the chain that causes the observed oscillation of heat pulse into the magnetic island. One is the onset of penetration of enhanced transport and a subsequent influence on the island width, through the modification of the thermal conductivity, which induces the two states in this system. The other is feedback on a longer time scale (the oscillation period between two states). The latter must be investigated in future, but is briefly commented on here. On the long time scale, the global magnetic shear, being the key in determining the width of the island, evolves in time. The mean magnetic shear influences the island width, so as to affect the two processes that are discussed in Section 3.2. When the *m* = 2 magnetic island is formed, the *m*/*n* = 0/0 current profile is influenced. The mean (0/0) bootstrap current is reduced near the mode rational surface, and is redistributed over the plasma column. This redistribution of mean current has much longer time scale than the time scale of redistribution of transport coefficient near the island. Thus, there are at least two distinctive time scales in conjunction with the on/off of penetration of heat pulse into the magnetic island.

## Discussion

In DIII-D during the application of an RMP field, the propagation of periodic heat pulses shows self-regulated oscillation between two states of a magnetic island: one shows low accessibility of the heat pulses and the other shows high accessibility of the heat pulses. It should be noted that this self-regulated oscillation occurs while the external C-coil perturbation field is held constant in time so this is represents a spontaneous bifurcation due to a change in plasma response. The state of small modulation amplitude of the heat pulse is interpreted as having nested surfaces resulting in a strong reduction of transport without turbulence penetration and the state of large modulation amplitude is interpreted as a high accessibility magnetic island due to the turbulence tunneling.

At this moment, the model in section 3.1 is the working hypothesis to investigate the observed transition in detail. The experimental observation that (1) the island width is larger than the calculation by use of vacuum field and (2) the response in the measured magnetic fluctuations has a delay of few ms when the transition occurs, seem to be consistent with the theoretical working hypothesis. In future, the local turbulence inside magnetic island should be measured in order to verify the cause of transitions.

This type of island can provide a strong poloidal asymmetry of radial heat flux because the transport inside the magnetic island is strongly reduced as observed in the much slower heat pulse propagates inside the island compared to that outside. Two states of the heat transport across a magnetic island can be explained by the hypothesis that turbulence spreading can occur in the island. This could trigger a self-regulated feedback oscillation of the island dynamics that is qualitatively consistent with the experimental observations. In one state the turbulence does not penetrate into the magnetic island, and the other the turbulence penetrates into magnetic island due to the process of turbulence tunneling.

Therefore, this experimental evidence of two states of a magnetic island gives a new insight into understanding the radial flux of (surface-averaged) heat transport with magnetic islands during RMP experiments. This understanding can be further improved and it may be possible to obtain better control of the heat transport in discharges with RMP fields by using the heat pulse propagation technique as a tool to identify the state of magnetic island.

## Methods

### DIII-D

DIII-D is a tokamak device with D-shape poloidal cross section, a major radius of 1.7 m and minor radius of 0.6 m for magnetic confinement of high temperature plasmas. In this experiment, the plasma current was 1.29 MA and the toroidal magnetic field is 1.97 T with an inner wall limiter configuration and a safety factor of *q*_95_ = 3.76. The line-averaged electron density was 3.35 × 10^19^ m^−3^ and the electron temperature in the core region was ~2 keV.

### Resonant magnetic perturbation (RMP)

Resonant magnetic perturbations (RMP) produced by a non-axisymmetric magnetic field perturbation coil (C-coil) is used to produce magnetic islands at the resonance surface where the safety factor is unity. In this experiment, the perturbation field has a resonance at *q* = 2 and the toroidal and poloidal mode number is 1 and 2, respectively. The toroidal phase of the C-coil is change by 180° to match the O-point or X-point of the magnetic island to the poloidal angle of the heat pulse measurements.

### Modulated electron cyclotron heating (MECH)

Heat pulse propagation experiments are a useful tool for identifying the magnetic topology in toroidal plasmas. In cases where the magnetic flux surface surfaces are stochastic, the heat pulse shows very fast propagation with large amplitude due to heat pulse propagation along the magnetic field on the time scale of thermal velocity. On contrast, in the region with a nested magnetic flux surface in the magnetic island, the heat pulse shows a bi-directional slow propagation with a small amplitude. The propagation speed of the heat pulse depends on the transport perpendicular to magnetic flux surface. When the transport inside the magnetic island is reduced due to the lack of turbulence, the propagation speed is expected to be extremely slow compared with that outside magnetic island. Therefore the magnetic topology (nested or stochastic) can be identified by measuring the amplitude and delay time of the heat pulse excited by MECH.

## Additional Information

**How to cite this article**: Ida, K. *et al.* Self-regulated oscillation of transport and topology of magnetic islands in toroidal plasmas. *Sci. Rep.* **5**, 16165; doi: 10.1038/srep16165 (2015).

## References

[b1] Wesson J. A. Magnetic island in Tokamaks 4th edn, 358–359 (Oxford, 2011).

[b2] Biskamp D. in Magnetic Reconnection in Plasmas Ch 8, 320–356 (Cambridge, 2000).

[b3] Shibata K. & Tanuma S. Plasmoid-induced-reconnection and fractal reconnection, Earth Planets Space 53, 473–482 (2001).

[b4] Evans T. E. *et al.* Edge stability and transport control with resonant magnetic perturbations in collisionless tokamak plasmas, Nature Phys. 2, 419–423 (2006).

[b5] Liang Y. *et al.* Active control of type-I edge-localized modes with n = 1 perturbation fields in the JET tokamak, Phys. Rev. Lett. 98, 265004 (2007). 17678097 10.1103/PhysRevLett.98.265004

[b6] Diamond P. H., Dupree T. H. & Tetreault D. J. Self-consistent model of stochastic magnetic fields, Phys. Rev. Lett. 45, 562–565 (1980).

[b7] Lichtenberg A. J., Itoh K., Itoh S.-I. & Fukuyama A. The role of stochasticity in sawtooth oscillations, Nucl. Fusion 32, 495–512 (1992).

[b8] Liang Y. *et al.* Observations of secondary structures after collapse events occurring at the q = 2 magnetic surface in the TEXTOR tokamak, Nucl. Fusion 47, L21–L25 (2007).

[b9] Ida K. *et al.* Topology bifurcation of a magnetic flux surface in magnetized plasmas, New J. Phys. 15, 013061 (2013).

[b10] Ida K. *et al.* Flow damping due to stochastization of the magnetic field, Nature com. 6, 5816; 10.1038/ncomms6816 (2015). PMC4308719 25569268

[b11] Ida K., Fonck R. J., Hulse R. A. & Leblanc B. Some effects of MHD activity on impurity transport in the PBX Tokamak, Plasma Phys. Controlled Fusion 28, 879–895 (1986).

[b12] Weller A. *et al.* Persistent density perturbations at rational-q surfaces following pellet injection in the Joint European Torus, Phys. Rev. Lett. 59, 2303–2306 (1987). 10035508 10.1103/PhysRevLett.59.2303

[b13] deVries P. C., Waidmann G., Krämer-Flecken A., Donné A. J. H. & Schüller F. C. Density profile peaking inside m/n=2/1 magnetic islands in TEXTOR-94, Nucl. Fusion 37, 1641–1646 (1997).

[b14] Inagaki S. *et al.* Observation of reduced heat transport inside the magnetic island O point in the large helical device, Phys. Rev. Lett. 92, 055002 (2004). 14995316 10.1103/PhysRevLett.92.055002

[b15] Ida K., Kamiya K., Isayama A. & Sakamoto Y. JT-60 Team, Reduction of Ion Thermal Diffusivity Inside a Magnetic Island in JT-60U Tokamak Plasma, Phys. Rev. Lett. 109, 065001 (2012). 23006274 10.1103/PhysRevLett.109.065001

[b16] Classen I. G. J. *et al.* Effect of heating on the suppression of tearing modes in tokamaks, Phys. Rev. Lett. 98, 035001 (2007). 17358689 10.1103/PhysRevLett.98.035001

[b17] Smolyakov A. I. Nonlinear evolution of tearing modes in inhomogeneous plasmas, Plasma Phys. Controlled Fusion 35, 657–687 (1993).

[b18] Kobayashi T. *et al.* Verification of wavelet analysis for a heat pulse propagation experiment, Plasma Phys. Control. Fusion 53, 095012 (2011).

[b19] Luxon J. L. A design retrospective of the DIII-D tokamak, Nucl. Fusion 42, 614–633 (2002).

[b20] Scoville J. T. & La Haye R. J. Multi-mode error field correction on the DIII-D tokamak, Nucl. Fusion 43, 250–257 (2003).

[b21] Ferraro N. M. *et al.* Role of plasma response in displacements of the tokamak edge due to applied non-axisymmetric fields, Nucl. Fusion 53, 073042 (2013).

[b22] Itoh K. *et al.* Self-sustained annihilation of magnetic islands in helical plasmas, Phys. Plasmas 12, 072512 (2005).

[b23] Hahm T. S. & Kulsrud R. M. Forced magnetic reconnection, Phys. Fluids 28, 2412 (1985).

[b24] Fitzpatrick R. Helical temperature perturbations associated with tearing modes in tokamak plasmas, Phys. Plasmas 2, 825–838 (1995).

[b25] Hahm T. S., Diamond P. H., Lin Z., Itoh K. & Itoh S.-I., Turbulence spreading into the linearly stable zone and transport scaling, Plasma Phys. Controlled Fusion 46, A323–A333 (2004).

[b26] Gurcan O. D., Diamond P. H., Hahm T. S. & Lin Z. Dynamics of turbulence spreading in magnetically confined plasmas, Phys. Plasmas 12 (2005) 032303

